# Pedestrian Detection Techniques for Advanced Driver Assistance Systems: A Comprehensive Review

**DOI:** 10.3390/jimaging12070317

**Published:** 2026-07-10

**Authors:** Dănuţ-Ovidiu Pop, Adrian-Silviu Roman

**Affiliations:** Electrical Engineering and Information Technology Department, George Emil Palade University of Medicine, Pharmacy, Science, and Technology of Targu Mures, 540142 Târgu Mureş, Romania; adrian.roman@umfst.ro

**Keywords:** pedestrian detection, advanced driver assistance systems (ADAS), computer vision, deep learning, intelligent transportation systems, multi-sensor fusion, real-time detection

## Abstract

Pedestrian detection is a fundamental component of Advanced Driver Assistance Systems (ADAS) and plays a key role in collision avoidance and the safety of vulnerable road users. This paper presents a structured review of pedestrian detection methodologies developed between 2000 and 2025, spanning classical vision techniques and modern deep learning architectures. We organize the review into two phases. First, we examine classical methods, including Histogram of Oriented Gradients (HOG)+Support Vector Machine (SVM), Viola–Jones, Deformable Part Models, and Integral Channel Features, which established the conceptual foundations of the field. Then, we analyze state-of-the-art deep learning architectures, categorized by detector stage (one-stage vs. two-stage), localization strategy (anchor-based vs. anchor-free), feature extraction paradigm (Convolutional Neural Network (CNN)-based vs. transformer-based), output representation (bounding box vs. instance segmentation), and computational profile (lightweight vs. heavyweight). Several design principles introduced by classical methods remain visible in modern architectures, indicating that they were not fully superseded. The review also examines publicly available benchmark datasets and compares the strengths and limitations of camera-, Light Detection And Ranging (LiDAR)-, radar-, and multi-sensor-fusion-based systems for ADAS deployment. We close by identifying six open problems for the field: adversarial robustness, real-time inference under embedded constraints, detection under adverse weather, dataset bias and demographic fairness, the deployment of Bird’s-Eye View (BEV) and unified perception on automotive hardware, and explainability for safety-critical use.

## 1. Introduction

Intelligent road safety systems have grown more capable, with the goal of making traffic safer and reducing the risk of accidents. A key part of ADAS is accurately detecting and classifying vulnerable road users, such as pedestrians, to prevent or reduce the severity of collisions [1,2].

Pedestrians represent one of the most vulnerable categories of road users, and pedestrian fatalities remain a significant public health issue. According to the World Health Organization (WHO), pedestrians accounted for 23% of the estimated 1.19 million global road traffic deaths reported in 2021 [3]. Smart mobility and autonomous driving systems have made pedestrian safety a fundamental requirement in the development of intelligent transportation systems [1,4]. The accurate and timely identification of pedestrians is essential, since it determines the response time of active safety measures such as autonomous braking and warning systems [2,4].

Over the past two decades, pedestrian detection research has undergone a significant transformation. Early approaches relied on classical computer vision techniques based on handcrafted features and traditional machine learning classifiers. In contrast, recent developments in deep learning have produced substantial gains in both accuracy and real-time inference. Architectures such as You Only Look Once (YOLO) [5], Faster Region-based Convolutional Neural Network (R-CNN) [6], and MobileNet [7] have demonstrated strong capabilities in identifying pedestrians within complex visual environments [8]. Several challenges remain, including the reliable detection of pedestrians in crowded scenes, heavy occlusions, and difficult lighting conditions [8,9]. Robustness across these conditions remains the central open problem.

Several questions remain about the evolution of pedestrian detection technologies. Although early computer vision approaches based on handcrafted features performed competitively in controlled environments, they are now often overlooked [8]. Several of their ideas remain relevant and could still be reused or extended in modern pedestrian detection models. With the emergence of deep learning, many of these methods have been replaced by data-driven architectures. This raises the following research question: are classical techniques obsolete, or do their underlying design principles persist inside modern architectures?

Although deep neural networks have become the dominant paradigm for pedestrian detection, several concepts introduced by classical computer vision (e.g., gradient-based feature extraction, region proposal strategies, multi-scale detection, and efficient post-processing mechanisms) continue to influence modern perception pipelines. Tracing how these ideas evolved helps explain the design choices behind current detectors.

**Figure 1:**
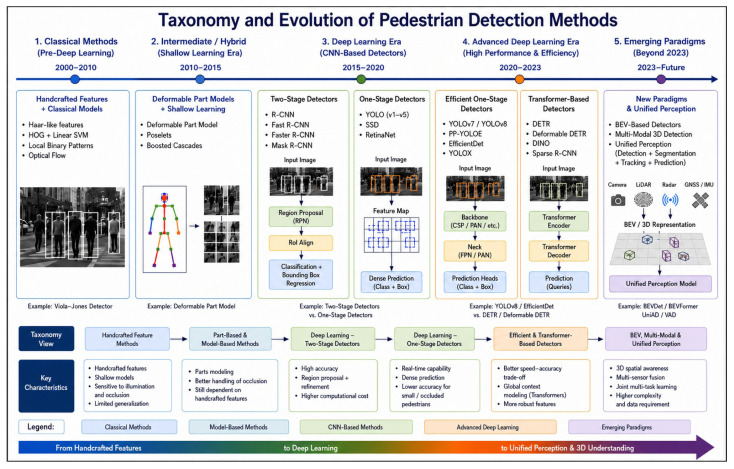
Evolution and taxonomy of pedestrian detection methods, from classical handcrafted approaches to modern deep learning architectures, including two-stage, one-stage, transformer-based, and BEV/unified perception frameworks.

To investigate this evolution, we adopt a dual-perspective analysis that contrasts classical and modern pedestrian detection paradigms. First, we examine classical vision-based pedestrian detection approaches developed between 2000 and 2020, focusing on representative handcrafted feature methods that shaped the conceptual foundations of the field. Second, we analyze modern deep learning-based pedestrian detection models published between 2020 and 2025, emphasizing the transition toward convolutional neural networks, transformer-based detectors, and multi-sensor perception architectures used in contemporary ADAS platforms. This temporal perspective allows us to highlight the technological progression of pedestrian detection and identify which design principles have persisted across different generations of detection systems (see Figure 1).

The main contributions of this review are summarized as follows:

- Longitudinal analysis of pedestrian detection technologies.
  We present a structured overview of the evolution of pedestrian detection methods, tracing the transition from classical computer vision techniques based on handcrafted features to modern deep learning-based detection architectures used in ADAS systems.
- Taxonomy across three dimensions: detector architecture (two-stage vs. one-stage), computational complexity (heavyweight vs. real-time), and sensing modality (vision-only vs. multi-sensor fusion).
- Comparative analysis of CNN-based and transformer-based detectors developed between 2020 and 2025, with attention to their suitability for real-time automotive deployment.
- Evaluation of widely used datasets and benchmarking practices, with attention to dataset bias, environmental variability, and the difficulty of real-world deployment.
- Identification of emerging research directions. We discuss recent developments such as multi-sensor fusion, 3D pedestrian detection, BEV-based perception, and unified perception frameworks, outlining key challenges and potential future directions for improving pedestrian detection reliability in ADAS systems.

The remainder of this paper is organized as follows. Section 2 introduces the methodological framework and taxonomy used to categorize pedestrian detection approaches. Section 3 reviews pedestrian detection techniques, covering classical handcrafted-feature methods, modern deep learning architectures organized along five design axes (detector stage, localization strategy, feature extraction, output representation, computational profile), and sensor approaches (camera, LiDAR, radar, and multi-sensor fusion). Section 4 examines publicly available benchmark datasets and evaluation metrics. Section 5 identifies open challenges and proposes directions for future research. Section 6 presents the conclusions.

## 2. Review Methodology and Structure

This section describes our review methodology and the categories we use to organize the literature. First, we review classical pedestrian detection methods based on handcrafted feature engineering [8,10,11]. Second, we analyze modern deep learning paradigms that employ neural network architectures to achieve higher detection accuracy and robustness in complex environments [8,12].

To ensure a structured and transparent process, we follow a PRISMA-inspired review protocol. Relevant publications were collected from major scientific databases including IEEE Xplore, Scopus, Web of Science, and Google Scholar. The literature search focused on peer-reviewed articles published between 2000 and 2025.

The search was conducted using combinations of the following keywords:

- Pedestrian detection.
- Vulnerable road user detection.
- ADAS perception systems.
- Deep learning pedestrian detection.
- Multi-sensor pedestrian detection.
- Object detection for autonomous driving.

To ensure the relevance and quality of the reviewed studies, the following inclusion criteria were applied:

- Studies addressing pedestrian detection in intelligent transportation systems or ADAS environments.
- Publications proposing or evaluating pedestrian detection algorithms based on classical computer vision or deep learning approaches.
- Studies reporting experimental evaluations on publicly available datasets.

Studies unrelated to pedestrian detection or lacking experimental validation were excluded from the analysis.

We categorize the reviewed methods along several design dimensions. First, detector architectures are classified into two-stage detectors, one-stage detectors, and more recent transformer-based models. Second, detection strategies are distinguished based on their localization mechanisms, including anchor-based and anchor-free approaches. Third, methods are evaluated according to their computational characteristics, differentiating between heavyweight detectors and lightweight models suitable for real-time deployment in embedded ADAS systems. Finally, the review considers the sensing modality employed by each approach, including vision-only systems and multi-sensor fusion frameworks.

## 3. Pedestrian Detection Techniques

### 3.1. Early Pedestrian Detection Techniques

**Figure 2:**
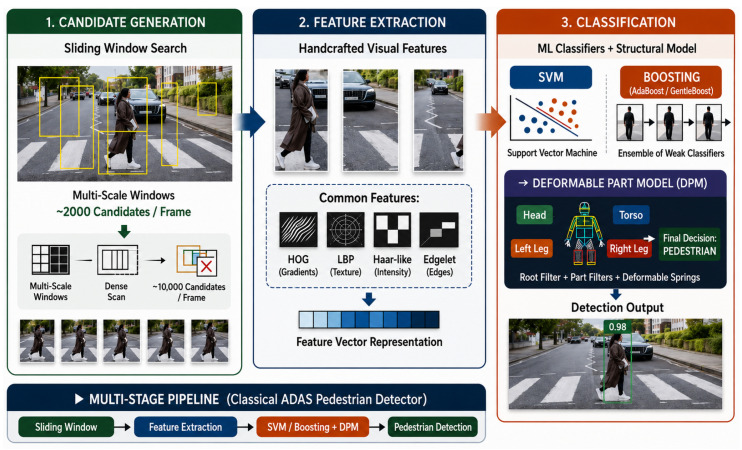
The early pedestrian detection approaches. Classical multi-stage pedestrian detection pipeline based on handcrafted features and traditional machine learning. The pipeline consists of three stages: (1) candidate generation using a multi-scale sliding-window search, producing approximately 2000 candidate regions per frame; (2) handcrafted feature extraction (e.g., HOG, LBP, Haar-like, and edgelet features) to generate feature vectors; and (3) pedestrian classification using SVM or boosting classifiers combined with a deformable part model (DPM). Yellow rectangles indicate candidate windows generated during the sliding-window search, blue arrows denote the processing flow between stages, the green bounding box indicates the final pedestrian detection, and the confidence score (0.98) represents the classifier confidence. Colored boxes in the DPM identify the modeled body parts (head, torso, left leg, and right leg) used for structural pedestrian representation. This paradigm forms the conceptual foundation for modern two-stage deep learning detectors.

Early pedestrian detection systems combined handcrafted visual features with classical machine learning classifiers. These systems usually followed a modular multi-stage pipeline: candidate region generation (typically via the sliding window technique), feature extraction, and classification using algorithms such as SVM or boosting-based methods (see Figure 2). To handle the articulated structure of the human body, structural models like the Deformable Part Model (DPM) were introduced to represent pedestrian geometry through a collection of part-based templates [13]. Before the emergence of deep CNNs [14,15,16], the field was defined by the exploration of robust descriptors such as Scale-Invariant Feature Transform (SIFT) [17], Local Binary Patterns (LBP) [18], Speeded-Up Robust Features (SURF) [19], HOG [20], and Haar-like features [21], which aimed to encode cues like texture, gradient orientation, and edge contrast.

Among these, HOG combined with a linear SVM (HOG+SVM) [20] became the leading approach for several years, with strong performance on local shape and gradient features. Dalal and Triggs [20] demonstrated that fine-scale gradients without smoothing, nine orientation bins over 0°–180°, and overlapping blocks with L2-Hys normalization were all critical for robust pedestrian representation, design principles that, as discussed below, continue to influence modern feature extraction pipelines. The Viola–Jones detector [21], which used Haar-like features and cascade AdaBoost, also offered efficient real-time detection, introducing three ideas that proved foundational beyond face detection: integral images for constant-time feature evaluation, AdaBoost-based automatic feature selection from a large pool of Haar-like candidates, and attentional cascades that rapidly discard background regions. These mechanisms directly prefigured the region proposal and early-rejection strategies used in modern two-stage detectors such as Faster R-CNN [6]. Another key group of methods used channel features, like Integral Channel Features (ICF), which built on HOG by adding more feature channels such as gradient magnitude and color, and used boosting for feature selection and classification [22,23]. These methods balanced accuracy against speed, and led to extensions like Aggregated Channel Features (ACF) [22] and filtered channel features [24]. Nam et al. [25] introduced Locally Decorrelated Channel Features (LDCF), demonstrating that applying learned decorrelation filters to ACF channels before boosted classification significantly reduced miss rate. This idea of transforming feature representations to reduce redundancy before classification foreshadows the normalization and decorrelation layers (e.g., batch normalization) prevalent in modern deep learning architectures.

At the same time, DPM [26,27] became popular for modeling pedestrian structure and dealing with pose changes and occlusions. DPM uses a root filter and several deformable parts, usually described with HOG features [20], and combines their responses while considering deformation costs. Variants of DPM improved results by using multi-resolution representations, handling occlusions, and adding context [28,29,30,31,32].

For inference, handcrafted features are taken from detection windows at different image scales, and classifiers separate pedestrians from the background. Non-Maximum Suppression (NMS) removes duplicate detections. In training, positive and negative samples are chosen based on their overlap with ground truth, and hard negative mining (bootstrap) [21] is often used to make detection more robust. Although these methods performed well, they struggled with large appearance changes and complex scenes, which led to the shift toward deep learning approaches [8].

These approaches were widely adopted because they ran in real time on the limited hardware of the period. As a result, they were suitable for early embedded vision systems used in intelligent transportation applications.

Handcrafted feature-based methods often struggled to generalize across complex real-world environments characterized by occlusions, variations in pedestrian appearance, and changing illumination conditions. Although classical techniques are no longer competitive with deep learning on detection accuracy, several of their underlying principles–such as gradient-based feature extraction, multiscale analysis, and efficient region evaluation–have influenced the design of contemporary deep learning detection architectures.

These limitations motivated the move to data-driven approaches. Deep learning has produced large gains in pedestrian detection accuracy by automatically learning hierarchical feature representations from large-scale datasets.

### 3.2. Deep Learning-Based Techniques

Deep learning has significantly advanced pedestrian detection by improving detection accuracy and robustness [9,33,34]. Unlike classical computer vision approaches that rely on handcrafted features, deep learning models automatically learn hierarchical feature representations directly from large-scale datasets [35]. The gradient-based local feature encoding introduced by HOG [20] is structurally similar to the first convolutional layers of modern CNNs, which also learn oriented edge and gradient filters [14]. This transition has resulted in substantial performance improvements, especially in complex environments encountered in ADAS. CNNs drove this progress by automatically extracting the discriminative features needed to detect pedestrians under challenging conditions such as occlusion, scale variation, and diverse lighting [36].

Modern deep learning detectors can be categorized along several architectural axes. These include the distinction between one-stage and two-stage detection frameworks, anchor-based and anchor-free detection strategies, CNN-based and transformer-based architectures, bounding box detection versus instance segmentation approaches, and lightweight versus heavyweight models designed for different deployment scenarios. The following subsections discuss these categories and highlight their respective strengths and limitations. A comparative summary is provided in Table 1.

To complement the qualitative taxonomy, Table 2 provides a quantitative comparison of representative RGB-only methods evaluated on the Caltech and CityPersons benchmarks, and Table 3 summarizes the multispectral/RGB-thermal methods, which are evaluated on different datasets (KAIST Multispectral Pedestrian Dataset (KAIST), Low-Light Visible-Infrared Paired dataset (LLVIP), MSDS) and report different metrics. Because these figures originate from heterogeneous sources, we treat Table 2 as a comparative literature summary rather than a controlled benchmark, and we characterise each method’s fitness for ADAS as explained further. A method is rated High when real-time operation (≥30 Frames Per Second (FPS), measured as single-image latency) has been demonstrated on embedded or edge hardware, or when a lightweight footprint (≤15 M parameters) is combined with real-time single-image latency on a desktop GPU. A method is considered Moderate when real-time operation is shown only on desktop or server GPUs, or is evidenced solely by batched or TensorRT throughput, which does not establish single-stream latency. Methods are rated Limited when they are not real-time under the reported conditions or when their computational footprint makes embedded deployment unlikely. Classical handcrafted methods retained for historical context are labelled Reference. Throughput figures cannot raise a rating above Moderate, since they do not characterise the per-frame latency that governs closed-loop safety. The rating reflects deployment feasibility under embedded compute constraints.

Several trends emerge from the RGB comparison. First, the progression from classical to deep learning methods is substantial: HOG+SVM [20] achieves a miss rate of 68.0% on Caltech, while the Adapted Faster R-CNN [59] lowers the Caltech log-average miss rate from 68.0% to roughly 5.0%. Second, the accuracy-speed trade-off between two-stage and one-stage detectors is reflected in the data: Faster R-CNN [6] achieves strong miss rates (∼15% on CityPersons Reasonable, vanilla configuration) but operates at only ∼5 FPS, whereas YOLOv8s [55] reaches ∼91 FPS with a comparable miss rate of ∼13.0%. Thus, YOLOv8s appears more favorable for latency-constrained deployment than two-stage alternatives under the reported conditions, although embedded validation remains necessary. Third, the lightweight variants show why a speed figure must be read together with its measurement type. Tiny-YOLOv3 runs at ∼21–45 FPS as single-image latency on an embedded Jetson Nano, which justifies its High suitability rating, whereas YOLOv8n’s headline ∼1010 FPS is batched TensorRT throughput on a server-class A100 rather than single-stream latency, so it does not by itself establish embedded real-time performance and is rated only Moderate. Finally, the table makes visible a fragmented evaluation landscape: most methods are tested on only one or two benchmarks under varying protocols and on different hardware, which limits direct comparability. Multispectral methods (Table 3) are not directly comparable to the RGB-only rows as they require aligned RGB and thermal input and are evaluated on dedicated benchmarks.

On the KAIST reasonable subset the classical baseline reaches 54.4% MR^−2^ while modern target-aware fusion methods achieve below 5% MR^−2^, indicating substantial progress within the multispectral approaches.

Table 2 reports a Heavy Occlusion column, and it is where the strong benchmark numbers come apart. The Adapted Faster R-CNN scores 12.97% on CityPersons Reasonable but 50.5% under heavy occlusion. CSP, at 11.0% on Reasonable, rises to roughly 49.3%. Each misses close to half the heavily occluded pedestrians it is shown. These are not edge cases for a driver-assistance system. For example, a child partially hidden between parked vehicles or a pedestrian embedded in a dense crowd represents precisely the kind of occluded case in which late detection has the highest safety cost. Read on their own, the Reasonable-subset figures flatter these methods, and the occlusion survey by Li et al. [69] finds the same gap across a wider set of detectors.

When most of a pedestrian is hidden, the features the network extracts look more like background than a person, and its confidence drops below the detection threshold. A second failure shows up in crowds: non-maximum suppression cannot tell a duplicate box on one pedestrian from a legitimate box on the person overlapping them, so it discards real detections alongside the redundant ones [32].

These issues are not new. Part-based models were built for exactly this difficulty. The occlusion-handling detector of Ouyang and Wang [70] treated a pedestrian as parts with their own visibility, so a hidden region cost one part rather than the whole detection. The methods that deal best with crowds and occlusion today have circled back to that idea, reasoning about visible regions explicitly, adding losses that penalise a box for drifting onto its neighbour, and letting suppression adapt to local crowd density [71,72,73]. Of the classical ideas this review traces into modern detectors, part-based occlusion reasoning is one of the clearest to have come back.

From an ADAS deployment perspective, the evolution of deep learning-based pedestrian detection is driven by accuracy improvements and by the ability to meet strict real-time and reliability constraints. While modern detectors report impressive benchmark performance, their behavior under real-world conditions—such as dense urban traffic, partial occlusion, and dynamic lighting—often reveals limitations. In particular, models optimized for benchmark datasets may fail to generalize when faced with domain shifts or sensor noise. Thus, a fundamental gap remains between academic evaluation and real-world deployment, suggesting that future research should prioritize robustness and adaptability over incremental accuracy gains.

#### 3.2.1. Two-Stage Detectors vs. One-Stage

**Figure 3:**
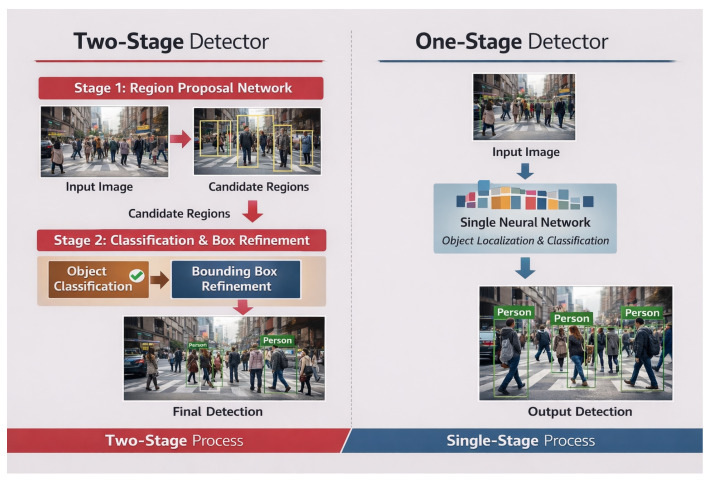
Comparison of two-stage and one-stage object detection architectures. The two-stage method first generates region proposals and then performs classification and bounding box refinement, while the one-stage method directly predicts object classes and locations in a single pass.

Deep learning object detectors are usually categorized into two-stage and one-stage architectures (see Figure 3). Two-stage detectors first generate candidate object regions via a region proposal network, then classify and refine them in a second stage. This paradigm has clear roots in classical detection methods: the cascade rejection principle introduced by [21], where a lightweight first stage rapidly eliminates background regions before a more expensive second stage processes the remaining candidates, can be seen as an early form of two-stage detection. More directly, the DPM framework [27] employed a coarse root filter to identify candidate locations and higher-resolution part filters to refine detections, a structure that is mirrored by the region proposal network and classification head in Faster R-CNN [6]. An important extension of this approach is the Discriminative DPM for pedestrian detection with occlusion handling, which explicitly models object parts and their spatial relationships to remain robust when portions of the pedestrian are not visible [70]. By allowing individual parts to be independently detected and spatially rearranged, this formulation improves detection performance in partially occluded scenarios, which are common in real-world urban environments.

Representative models in the modern two-stage category include R-CNN, Fast R-CNN, Faster R-CNN [6], and Mask R-CNN [38]. These approaches generally achieve high detection accuracy, largely due to their region-wise processing mechanism, which is detailed below.

The sequential region proposal and classification process introduces additional computational overhead, which limits their applicability in real-time applications with constrained computational resources [39,40].

One-stage detectors localise and classify objects in a single forward pass, without a separate region proposal stage. Prominent examples include YOLO [5] and Single Shot MultiBox Detector (SSD) [45]. These models are optimized for high-speed inference and are therefore widely used in real-time applications such as ADAS perception systems. However, one-stage detectors typically exhibit reduced accuracy when detecting small or densely packed objects [37,42].

The performance differences between the two approaches are rooted in their internal processing mechanisms. In particular, two-stage detectors rely on a Region Proposal Network (RPN) that generates candidate regions by sliding a small convolutional network over the feature map and predicting objectness scores together with bounding box offsets for a set of predefined anchors. These proposals are subsequently refined in a second stage using Region of Interest (RoI) operations, which extract fixed-size feature representations for precise classification and localization. This region-wise processing enables more accurate bounding box regression, particularly for small or partially occluded pedestrians, as feature extraction is focused on a reduced set of candidate regions.

In contrast, one-stage detectors apply detection as a dense prediction problem, where bounding boxes and class probabilities are directly regressed at each spatial location of the feature map in a single forward pass. As a consequence of this design, the model must learn object localization and classification simultaneously across all spatial locations, increasing sensitivity to background noise. While this approach eliminates the computational overhead associated with proposal generation, it introduces a class imbalance problem, since the majority of spatial locations correspond to the background. This imbalance can negatively affect training stability and detection accuracy, especially for small objects. Because predictions are tied to fixed-resolution feature maps, small pedestrians may not be sufficiently represented, leading to reduced localization precision compared to two-stage approaches.

This trade-off between detection accuracy and computational efficiency is important in ADAS environments, where maintaining high frame rates is critical for real-time safety systems [43,44]. The speed–accuracy trade-off alone is insufficient to fully characterise detector performance in such contexts. Instead, the suitability of a detection architecture depends strongly on the operational scenario. For example, highway scenes with low object density may tolerate slower two-stage detectors, whereas dense urban traffic requires faster one-stage approaches.

#### 3.2.2. Anchor-Based vs. Anchor-Free Detectors

Object detection models used in pedestrian detection for ADAS can be broadly categorized based on their bounding box prediction strategy. Anchor-based detectors rely on predefined anchor boxes with multiple scales and aspect ratios to localize pedestrians. These anchors are matched to ground-truth bounding boxes during training, enabling the model to learn localization through regression. Widely used architectures such as Faster R-CNN [6], SSD [45], and RetinaNet [74] have been successfully applied to pedestrian detection benchmarks such as Caltech and CityPersons.

Real-world driving presents three specific challenges: scale variation (far vs. near pedestrians), frequent occlusions, and dense crowds in urban environments. Anchor-based methods require careful tuning of anchor sizes and aspect ratios to match the upright, narrow shape of pedestrians. In practice, suboptimal anchor design can lead to poor matching quality, especially for small or heavily occluded pedestrians, which are critical cases in ADAS safety systems [75].

Anchor-free detectors avoid this by removing predefined anchors entirely. Instead, these models directly predict object centers or keypoints and regress bounding box dimensions. Approaches such as CenterNet [75] and FCOS [62] simplify the detection pipeline and reduce the number of hyperparameters, making them attractive for real-time ADAS applications where robustness and efficiency are crucial.

In pedestrian detection, anchor-free methods offer several advantages. First, they handle scale variation more naturally by assigning predictions to spatial locations rather than predefined anchor boxes. Second, they improve detection in crowded scenes by reducing ambiguity in anchor matching, which is a common issue when multiple pedestrians overlap. For example, FCOS formulates detection as a per-pixel prediction task, allowing better localization of small and partially occluded pedestrians. Similarly, CenterNet models pedestrians as center points, which has been shown to improve performance in dense urban traffic scenarios [47,62].

Recent work in ADAS-oriented pedestrian detection has also explored hybrid and improved strategies. Methods such as Adaptive Training Sample Selection (ATSS) [46] reduce the sensitivity of anchor-based detectors by dynamically selecting positive samples, while newer detectors like YOLOX [76] adopt anchor-free formulations to improve both speed and accuracy. These approaches trade detection precision against computational cost, which is the binding constraint for embedded automotive deployment.

Anchor-based detectors remain widely used because they are mature and well-supported. However, anchor-free methods are increasingly the more flexible choice. Their ability to better handle small, occluded, and densely packed pedestrians makes them suitable for safety-focused driving scenarios, where accurate and reliable detection is essential.

From a technical perspective, the key difference lies in how object localization is performed. Anchor-based methods treat detection as a matching and regression problem over predefined hypotheses, where performance depends on the quality of anchor design and assignment. In contrast, anchor-free methods reformulate detection as a spatial prediction task, directly learning object locations from feature representations, which simplifies the pipeline but shifts complexity toward accurate feature learning.

#### 3.2.3. CNN-Based vs. Transformer-Based

CNNs have long been the dominant approach for pedestrian detection in ADAS due to their efficiency in extracting local spatial features [60] and enabling real-time performance on embedded systems [37].

Transformer-based models take a different approach, using self-attention to capture global contextual relationships across the entire scene [49]. This is valuable for pedestrian detection, where scene context—object interactions, occlusions, and environmental structure—improves detection accuracy.

Recent transformer-based architectures such as DEtection TRansformer (DETR) [50] and Swin Transformer [77] apply global self-attention to capture the scene context relevant to small or partially occluded pedestrians in complex urban environments. The benefit is not unconditional, as large-scale cross-dataset studies report that CNN backbones still generalise better than transformer backbones under domain shift [78,79]. Additionally, hybrid models such as Real-Time DEtection TRansformer (RT-DETR) [63] integrate transformers with CNN backbones to enhance feature representation while maintaining computational efficiency [61]. These approaches combine the strengths of CNNs (efficient local feature extraction) with those of transformers (global context modeling).

The adoption of transformers in ADAS is constrained by their computational cost. The self-attention mechanism scales quadratically with input size, making real-time inference challenging on resource-limited hardware [51,80]. This limitation is critical in safety-sensitive ADAS applications, where low latency and reliability are essential.

To address these challenges, recent research has focused on improving transformer efficiency through a range of architectural optimizations that extend beyond basic local attention and hierarchical processing. In addition to techniques such as local attention, hierarchical feature processing, and input downsampling—used in models like Swin Transformer [77] and Pyramid Vision Transformer (PVT) [81]—more advanced attention optimization strategies have been proposed to further reduce computational complexity while preserving global contextual modeling [82,83].

In particular, local attention mechanisms limit the self-attention operation to small, fixed regions of the image instead of comparing every location with all others. This means the model focuses only on nearby areas at a time, which reduces the amount of computation while still capturing important local patterns [77]. Hierarchical processing further improves efficiency by progressively reducing spatial resolution while increasing feature abstraction, allowing global context to be captured across layers instead of within a single attention operation [81].

Beyond these approaches, sparse attention mechanisms reduce computational cost by limiting interactions to a subset of relevant tokens instead of computing full pairwise relationships, lowering memory and processing requirements [84,85]. Deformable attention, as introduced in Deformable DETR, further enhances efficiency by learning a small set of sampling points around reference locations, enabling the model to focus on the most informative spatial regions while reducing unnecessary computations [86]. Additionally, linearized attention methods approximate standard attention operations to achieve linear complexity (O(N)), enabling scalability to high-resolution inputs typical in ADAS scenarios [87].

Input downsampling also plays a critical role by reducing the number of tokens processed by the transformer, directly lowering computational cost, although at the potential expense of fine spatial detail [83]. These strategies collectively enable transformer models to approximate global reasoning while remaining computationally feasible for real-time ADAS applications.

Hybrid CNN–Transformer architectures combine efficient convolutional feature extraction with transformer-based global reasoning, allowing models to operate at reduced resolution while maintaining strong contextual awareness [50,63]. Alongside these developments, lightweight and edge-optimized vision transformers are being designed to meet the strict latency, memory, and energy constraints of embedded automotive systems [88].

Transformer networks now appear in higher-level tasks such as pedestrian intention prediction and trajectory forecasting, where modeling temporal dependencies in video sequences provides advantages in anticipating human behavior [89,90]. This highlights their broader potential in enhancing situational awareness in ADAS.

Robustness, explainability, scale variation, and occlusion remain unsolved in real-world scenarios [69,91].

CNN-based models remain the preferred choice for real-time pedestrian detection in ADAS due to their efficiency and lower computational requirements. In contrast, transformer-based models offer superior contextual understanding and improved performance in complex scenarios, such as occlusion and dense environments. The downside is the higher computational cost. Therefore, hybrid CNN–transformer architectures and optimized transformer designs represent a promising direction for achieving an effective balance between accuracy and real-time performance in practical ADAS deployments [92,93].

#### 3.2.4. Bounding Box Detection vs. Instance Segmentation

In ADAS, pedestrian detection has usually relied on bounding box-based object detectors, which estimate the location of pedestrians using rectangular regions. Approaches such as Faster R-CNN [6] and YOLO-based models [60] are widely used due to their computational efficiency and ability to operate in real time on embedded systems.

Bounding boxes provide only coarse localization, which is insufficient in complex driving scenarios, such as crowded urban environments or partial occlusions. This limitation is critical in ADAS, where precise understanding of pedestrian shape and position directly impacts safety and collision avoidance.

To address these challenges, pedestrian instance segmentation methods have been increasingly adopted. These approaches generate pixel-level masks for each pedestrian, enabling accurate delineation of object boundaries. Models such as Mask R-CNN [38,54], YOLACT [94], and SOLOv2 [95] provide more detailed spatial information, which is essential for handling occlusions, distinguishing closely spaced pedestrians, and improving detection in dense traffic scenes.

In ADAS applications, this fine-grained segmentation plays an important role in higher-level tasks such as pedestrian intention prediction and trajectory estimation. Silhouette-level masks let these systems infer motion, body orientation, and interactions more accurately [96,97].

Segmentation’s precision comes at a cost, as pixel-level prediction demands more compute and memory, which makes real-time performance hard on resource-constrained automotive hardware. Segmentation models are sensitive to real-world conditions such as illumination changes, weather variations, occlusion, and viewpoint diversity, which can degrade performance in practical ADAS deployments [53,98].

To overcome these limitations, recent research in pedestrian segmentation for ADAS focuses on improving both efficiency and robustness. Lightweight real-time models such as YOLACT [94] aim to reduce computational cost, while transformer-based segmentation approaches like Mask2Former [99] enhance robustness by incorporating global contextual information.

Modern ADAS systems increasingly rely on multi-modal perception to improve pedestrian segmentation accuracy. By integrating data from cameras, LiDAR, and radar sensors, these systems can refine object boundaries and maintain reliable detection under challenging conditions such as low lighting or heavy occlusion [100,101]. Sensor fusion techniques enable complementary strengths across modalities, improving robustness compared to vision-only approaches.

Bounding box detection remains the default for real-time performance, but lacks the precision required for safety-critical scenarios. Instance segmentation provides pixel-level masks that help with occlusion handling and trajectory inference, at higher compute cost.

#### 3.2.5. Lightweight vs. Heavyweight Models

A further design axis is the trade-off between detection accuracy and computational cost. Lightweight models, such as Tiny-YOLO and MobileNet-SSD, are specifically designed for deployment on resource-constrained embedded platforms commonly used in ADAS systems.

These architectures prioritize fast inference speed and reduced memory usage, enabling real-time operation on edge devices. In contrast, larger models, including full-scale YOLO variants and transformer-based detectors, often achieve higher detection accuracy but require significantly greater computational resources.

Selecting an appropriate detection model therefore requires balancing detection accuracy, inference speed, and available hardware capabilities. This trade-off is critical in autonomous driving scenarios, where timely detection of pedestrians is essential for safe vehicle operation [56,57].

### 3.3. Camera-Based Systems

Cameras capture color, texture, and shape, which helps separate pedestrians from background clutter. These systems employ advanced computer vision techniques, frequently using deep neural networks, to detect and classify pedestrians [61], and in some cases estimate pedestrian pose [2]. However, their performance is adversely affected by variable lighting conditions (including sunlight, shadows, and nighttime), inclement weather (such as rain, fog, and snow), and occlusion [68]. For instance, cameras may struggle to detect pedestrians at night or during heavy rainfall [9,102]. The inherent two-dimensional nature of camera imagery complicates accurate distance estimation, which is critical for collision avoidance. To address these challenges, emerging techniques such as multi-exposure imaging and advanced image enhancement are being developed to improve detection under challenging lighting conditions [103].

### 3.4. LiDAR-Based Systems

LiDAR-based systems provide highly accurate 3D information by emitting laser pulses and measuring their return time, creating detailed maps of the environment [33]. Because LiDAR measures depth directly, it is less affected by changes in lighting and the appearance variation that degrades camera-based detection [104]. However, LiDAR sensors are costly, degrade under heavy rain or fog, and produce point-cloud bandwidth that demands non-trivial onboard compute [105]. Even with these drawbacks, precise 3D geometry supports pedestrian classification and accurate localization in range and velocity, which is important for ADAS [106]. LiDAR excels at 3D mapping, but it may not work as well for small pedestrians or in some weather conditions [107]. Because LiDAR is an active sensor, it retains its detection capability under low-light conditions that compromise camera-based pipelines [108]. Still, the usual angular resolution of LiDAR (0.1° to 0.4°) can limit detail, especially for small or far-away pedestrians, compared to the higher resolution of cameras [4]. So, while LiDAR gives accurate depth, the shapes of pedestrians may not be as clear, and advanced point cloud processing is needed for reliable detection [109,110].

### 3.5. Radar-Based Systems

Radar-based systems complement other sensing modalities, as radar is robust to adverse weather and measures radial velocity directly via the Doppler shift, supporting pedestrian tracking despite lower spatial resolution than cameras or LiDAR [4]. Radar maintains detection range under fog and heavy rain, conditions in which cameras typically fail [111,112]. Modern 77-GHz radar systems are accurate for pedestrian detection in good conditions and are used in adaptive cruise control, while 24-GHz radar is being studied for closer ranges [109,113]. The main drawback is their low spatial resolution, which makes it hard to tell individual pedestrians apart or see their exact shape in crowds [114]. Radar is therefore typically fused with cameras, contributing weather robustness and velocity, while cameras contribute spatial detail [115]. This combination improves detection accuracy and reduces false positives [9,116]. Detecting occluded pedestrians reliably still requires either learned radar representations or multi-sensor fusion [117].

### 3.6. Fusion-Based Systems

**Figure 4:**
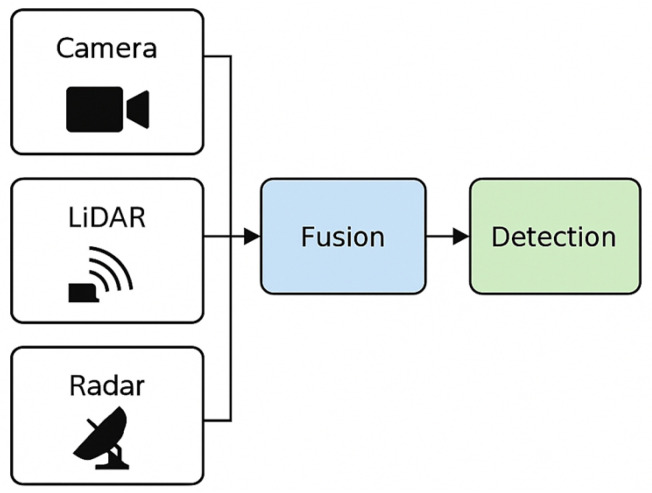
Multi-sensor fusion architecture combining camera, LiDAR, and radar data for robust pedestrian detection.

**Figure 5:**
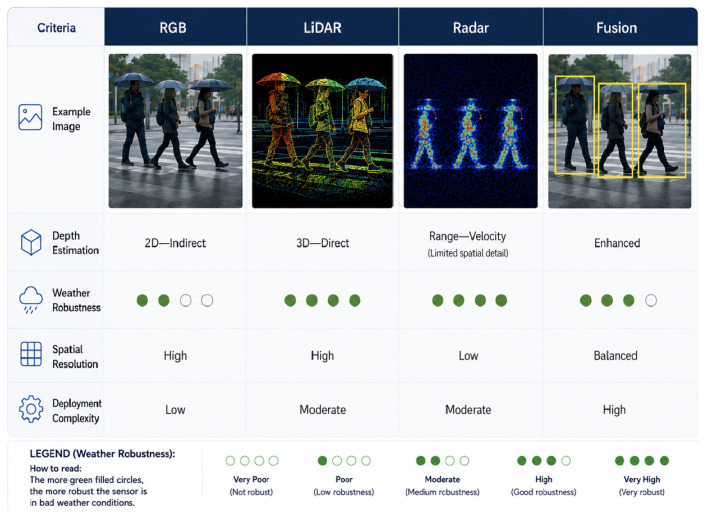
Comparison of sensing modalities for pedestrian detection. Red, Green, Blue (RGB) cameras provide high spatial resolution but rely on indirect depth estimation and are sensitive to adverse weather conditions. LiDAR enables accurate 3D structural perception with strong robustness but at moderate system complexity. Radar offers reliable range–velocity measurements under challenging environmental conditions, albeit with limited spatial resolution. Sensor fusion integrates complementary information from multiple modalities, resulting in enhanced detection accuracy and robustness at the expense of increased computational and calibration complexity.

Multi-sensor fusion combines data streams from complementary sensors to mitigate single-modality failure modes (see Table 4), giving a clearer and more complete view of the environment for better pedestrian detection [118]. This often means using radar, LiDAR, and cameras together (see Figure 4), exploiting modality-specific strengths to maintain detection under degraded conditions [4,119]. For example, combining camera and LiDAR provides both detailed images and accurate 3D locations, while radar-camera fusion adds weather robustness to camera-based detection [120]. Fusion improves reliability under single-sensor failure (see Figure 5) [121,122]. Newer methods, like conditional dynamic sensor fusion, change how much each sensor is used depending on the environment and confidence, using high-resolution 4D radar and LiDAR’s range resolution to improve detection under degraded conditions [123]. This method uses radar for long-range and speed, and cameras for detailed context, which helps find small objects like riders and bicycles [124]. However, these systems still struggle to detect fully occluded pedestrians, since current fusion methods mostly work for partially hidden cases and fail when no sensor has line-of-sight to the pedestrian [125].

The effectiveness of multi-sensor fusion depends on how information from different modalities is integrated within the detection pipeline. Fusion strategies are typically categorized into early fusion, late fusion, and feature-level fusion. In early fusion, raw sensor data or low-level representations are combined before feature extraction, allowing the model to learn joint representations across modalities. However, this approach is highly sensitive to spatial misalignment, since even small calibration errors between sensors can lead to incorrect feature correspondence.

In contrast, late fusion combines predictions from independently processed sensor streams at the decision level. While this improves robustness to sensor noise and misalignment, it limits the ability of the model to exploit cross-modal feature interactions.

Feature-level fusion represents a compromise, where intermediate feature maps from different modalities are combined during the network forward pass. This enables partial cross-modal interaction while maintaining greater robustness than early fusion. However, it introduces additional architectural complexity and requires careful spatial alignment between modalities.

A key challenge across all fusion strategies is the alignment of heterogeneous sensor data. Camera images, LiDAR point clouds, and radar signals operate in different coordinate systems and resolutions, requiring precise calibration and synchronization to ensure consistent feature integration. This highlights that the performance of multi-sensor systems is not determined only by the choice of sensors, but also by the effectiveness of the fusion strategy used to integrate their information.

The early, late, and feature-level split corresponds to real systems that differ mainly in how they reconcile sensors with different coordinate frames. PointPainting [126] is an early-fusion method: it labels each LiDAR point with the class of the camera pixel it projects onto, using the calibration matrix, and detects on the augmented cloud. The projection is fixed, so a few centimetres of extrinsic error paint points with the wrong labels, and the result is only as reliable as the calibration. CenterFusion [127] associates radar and camera in a similarly rigid way, attaching radar returns to image detections inside a frustum set by the camera geometry.

Feature-level methods relax this. Instead of an exact pixel-to-point match, TransFuser [128] lets camera and LiDAR features attend to each other through cross-attention, which tolerates the calibration drift a vehicle accumulates in service. Another line of work sidesteps correspondence entirely by lifting each modality into a bird’s-eye-view grid before fusing: BEVFusion [129] does this for camera and LiDAR, and MetaOcc [124] brings 4D radar into the same top-down frame. BEV also fits the rest of the stack, since planning and control already work in that frame, so the fused output needs no reprojection.

These results are sensitive to the evaluation setup. The 14% gain in pedestrian AP reported for low-resolution LiDAR fused with Doppler radar [116] came from fixed roadside sensors with no ego-motion. A vehicle-mounted system has to cope with calibration that shifts under vibration and load, and to compensate for its own movement, conditions a roadside rig never sees.

**Table 4:** Comparison of sensor modalities for pedestrian detection in ADAS systems.

Dimension	Camera	LiDAR	Radar	Multi-Sensor Fusion
Depth/3D info	Estimated (stereo or monocular); accurate distance estimation difficult [61]	Native 3D; environment maps via laser time-of-flight [33]	Doppler provides velocity only; no spatial shape information [4]	Camera + LiDAR: detailed images combined with accurate 3D locations [4,120]
Night/low-light performance	Light-dependent; Sunlight, shadows, and nighttime all degrade performance [68]	Light-independent operation; geometric data assists low-light detection [104]	Fully light-independent	Radar or thermal modality compensates for camera failure in darkness [118]
Adverse weather performance	Rain, fog, and snow reduce contrast and obscure visual detail [9,102]	Heavy rain and fog attenuate laser returns [105]	Penetrates fog and heavy rain [111,112]	Maintains detection when cameras fail [120,122]
Spatial resolution	High; rich color, texture, and shape information [61]	Typical 0.1°–0.4° angular resolution; limits detail for small or distant pedestrians [4,130]	Low; cannot resolve individual pedestrian shapes or separate crowds [114]	Combined spatial detail from complementary sensors [119]
Velocity measurement	Derived; computed from optical flow between successive frames; not a primary sensor output	Derived; estimated from scan-to-scan point cloud differencing; not a primary sensor output	Native; instantaneous radial velocity from Doppler frequency shift [4]	Native radar Doppler velocity supplements camera and LiDAR tracking [124]
Full occlusion handling	Hidden pedestrians missed entirely [68]	Point cloud may capture occluded geometry [108]	Velocity signal may persist behind obstacles [117]	Current methods mainly handle partial occlusion; fully hidden pedestrians remain an open problem [125]
Cost/deployment complexity	Low; widely deployed; minimal hardware overhead	High; expensive hardware; data-intensive processing requires strong computing power [105]	Moderate; 77-GHz systems embedded-ready; used in adaptive cruise control [109]	High; requires sensor calibration, synchronization, and compute overhead [118]
Mitigation	Multi-exposure imaging and image enhancement improve detection under challenging lighting conditions [103]	Advanced point cloud processing needed to interpret pedestrian shapes; depth information alone is insufficient [109]	Commonly combined with cameras to compensate for low spatial resolution and to exploit Doppler velocity [115,116]	Fusing velocity-encoded radar with low-res LiDAR improves pedestrian AP by 14% vs. single-sensor baseline [116]

## 4. Datasets and Evaluation Metrics

Multi-sensor pedestrian detection requires large and varied datasets that reflect real driving conditions [33]. Coverage of varied lighting, weather, and pedestrian behavior is required for models to generalize [4,105]. It is important to include data from different sensors, like LiDAR point clouds, radar Doppler data, and camera images, for training and testing fusion-based pedestrian detection systems [8]. For instance, datasets like CVC-14 and KAIST, which have both thermal and visible images, are helpful for testing detection under different lighting, while the nuScenes dataset offers a wide range of multi-sensor data for autonomous driving research [131].

### 4.1. Publicly Available Datasets

Building and evaluating pedestrian detection systems depends on publicly available datasets that capture the complexity of real driving. Over the past ten years, several benchmark datasets for pedestrian detection (summarized in Table 5) have become standard tools for research.

Among the most influential pedestrian-specific benchmarks is the Caltech Pedestrian Dataset [132], which contains extensive video sequences captured from a vehicle-mounted camera in urban environments. It provides detailed annotations of pedestrians under varying scales, levels of occlusion, and motion patterns, and has served as a foundational dataset for evaluating both classical and deep learning-based pedestrian detectors. Similarly, the CityPersons dataset [59], built on top of the Cityscapes dataset [133], offers high-quality bounding box annotations for pedestrians in complex urban scenes, emphasizing dense crowds, partial occlusions, and diverse viewpoints commonly encountered in ADAS scenarios.

The KITTI Pedestrian Dataset [134], part of the KITTI Vision Benchmark Suite, is another popular benchmark. KITTI offers synchronized stereo camera and LiDAR data collected from a moving vehicle in urban, rural, and highway environments. The pedestrian part of the dataset is used to test detection and localization in real driving conditions, especially when there are changes in size, motion blur, or partial occlusion. Its use of multiple sensors and standard evaluation methods has helped link academic research to real-world ADAS applications.

To help models work well in different places and conditions, the EuroCity Persons dataset [135] was created using data from several European cities in various weather and lighting. This dataset adds scene diversity and is now a standard for testing how robust pedestrian detection is in real driving. The WiderPerson dataset [136], by contrast, focuses on very crowded scenes with lots of occlusion, making it a tough test for modern pedestrian detection models.

Multispectral pedestrian datasets help address the limitations of visible-light cameras in low-light or nighttime conditions. The KAIST Multispectral Pedestrian Dataset [67] and the CVC-14 dataset [137] provide aligned visible and thermal infrared images, allowing researchers to test multispectral fusion strategies. These datasets are especially useful for evaluating pedestrian detection in nighttime and poor lighting, which is important for ADAS safety.

In addition to pedestrian-specific benchmarks, several large-scale autonomous driving datasets provide rich multimodal sensory data for fusion-based pedestrian detection research. Datasets like nuScenes [131], AIODrive [138], and Oxford Radar RobotCar [139] include synchronized data from cameras, LiDAR, radar, Global Positioning System (GPS), and inertial sensors. Although these datasets are not focused only on pedestrians, they offer broad sensor coverage and diverse driving conditions, making them useful for evaluating multi-sensor perception systems and studying pedestrian detection in wider traffic contexts [140].

Recently, event-based vision datasets have been created to help develop event-driven pedestrian detection methods. These datasets use neuromorphic vision sensors that record changes in light intensity as they happen, offering benefits like high dynamic range, low delay, and less motion blur, especially in tough lighting [141]. However, there are not many large, public event-based datasets just for pedestrian detection. Many current datasets have low resolution, few labels, or limited variety in environments, which makes them less useful for full ADAS testing [142]. Because of this, synthetic event data are often added to real recordings, but this can cause domain gaps and bias that need careful handling [143].

### 4.2. Performance Metrics

Pedestrian detection systems are evaluated with several performance metrics covering accuracy, reliability, and real-time behavior. Important metrics include precision, recall, F1-score, and mAP, which together show how well a detector finds pedestrians and avoids false alarms or missed detections at different overlap thresholds [79]. The most widely adopted evaluation metric on the Caltech and CityPersons benchmarks is the MR^−2^, which is computed as the log-average miss rate over the FPPI range $\left[10^{-2},\;10^{0}\right]$ [25,132]. Lower MR^−2^ values indicate better detection performance. This metric is used throughout the quantitative comparison presented in Table 2. Other key metrics, such as processing time, power use, and frames per second, are also important for seeing if these systems can work in real cars with limited resources [110]. It is also important to measure how well detection works in different conditions, such as changes in light, bad weather, and different levels of occlusion, since these affect safety and reliability in self-driving cars [4,144]. Beyond the usual metrics, recent work has begun reporting per-subgroup accuracy, motivated by documented disparities in detection rates across demographic groups [9,145]. Because of this, fairness-aware evaluation protocols stratified by age, apparent gender, and skin tone are an active area of method development.

A critical limitation in current pedestrian detection research is the lack of standardized benchmarking protocols across studies. As shown in Table 2, performance results are often reported on different datasets, under varying experimental conditions and hardware configurations, making direct comparison challenging. This fragmentation limits the ability to draw definitive conclusions about the superiority of specific methods. Consequently, there is a need for more unified evaluation frameworks that incorporate not only accuracy metrics but also real-time performance, robustness under adverse conditions, and deployment feasibility in ADAS systems.

## 5. Challenges and Future Directions

A key observation emerging from this review is that many of the remaining challenges in pedestrian detection are system-level in nature. Issues such as real-time processing constraints, sensor reliability, and environmental variability cannot be fully addressed by improving model architectures alone. Instead, they require integrated solutions that combine advances in hardware design, sensor fusion, and adaptive learning strategies. This shift from model-centric to system-centric design represents another direction for future ADAS research, where robustness and scalability take precedence over isolated performance improvements.

Despite progress in pedestrian detection for ADAS, several open problems block reliable deployment. We group them into six areas (Table 6): adversarial robustness, real-time inference under embedded constraints, detection under adverse weather, dataset bias and demographic fairness, the emerging BEV and unified-perception paradigms, and the need for explainable models in safety-critical settings.

### 5.1. Adversarial Attacks and Robustness

Another critical aspect is the vulnerability of deep learning models to adversarial attacks, where subtle perturbations to input data can lead to catastrophic misclassifications, posing significant safety risks in autonomous driving [147]. These vulnerabilities necessitate the development of robust detection models capable of maintaining performance against malicious alterations, highlighting the need for comprehensive testing methodologies that go beyond conventional performance benchmarks [146]. Addressing the biases of existing pedestrian detectors towards specific age or gender groups, especially during nighttime conditions, is essential for fostering equitable safety outcomes for all vulnerable road users [145]. This includes evaluating fairness across diverse demographics and environmental conditions to ensure that pedestrian detection systems do not inadvertently compromise the safety of certain groups [145,160].

### 5.2. Real-Time Processing and Efficiency

Real-time processing is essential for effective ADAS, so systems must be optimized for quick responses on embedded hardware while also being efficient to save power. This is important for fast reactions needed to avoid collisions and ensure safety in dense traffic. Additionally, it often means balancing detection accuracy with how much computing power is used [148]. Ongoing work on lightweight models and efficient processing engines, aims to ensure ADAS can detect pedestrians quickly and accurately without using too many resources [149]. More research is also needed into special hardware and new methods, like event-based vision systems, which can collect and process data with very low delay, improving real-time performance for pedestrian detection in ADAS [141]. Further improvements require new ways to combine data from different sensors, such as radar, LiDAR, and thermal cameras, to improve detection robustness, particularly under adverse conditions. This approach helps overcome problems caused by changes in brightness and contrast, which often affect systems that use only one type of sensor [145]. In addition, new Artificial Intelligence (AI) models like Vision Transformers, large language models, and diffusion models are being studied to improve how systems understand and predict behavior in complex traffic [9].

### 5.3. Detection in Adverse Weather Conditions

Adverse weather conditions, such as heavy rain, snow, fog, and glare can make vision-based pedestrian detection systems much less effective by hiding important visual details and lowering contrast, which leads to more missed detections and less safety [150]. To deal with this, better sensor fusion methods and stronger image enhancement techniques are needed to maintain detection accuracy under adverse conditions [9,141]. For example, the Illumination and Temperature-aware Multispectral Network can change how much it uses RGB and infrared features, relying more on thermal images in the dark [150]. New event-based cameras are also helping with extreme lighting changes, offering high dynamic range and very fast response times, which are important for detecting pedestrians in quickly changing weather [141]. To fix the problem of low-resolution images from regular cameras in dim light, researchers have evaluated Faster R-CNN and related detectors with noise augmentation under realistic weather scenarios [53]. Combining RGB and thermal infrared images, known as multispectral fusion, is now a common way to improve detection, especially when contrast is low and noise is high [9,36].

### 5.4. Ethical Considerations and Bias

Combining data from different sources can help address challenges such as detecting smaller pedestrians and estimating their motion relative to the vehicle, which are difficult tasks for single-camera systems [161]. This fusion improves detection accuracy and robustness in challenging conditions, such as poor lighting or occlusions, and can partially mitigate issues related to limited or imbalanced datasets [152].

However, despite these technical advantages, important ethical concerns remain. A significant source of bias originates from the datasets used to train these systems. Many publicly available image datasets do not adequately represent all categories of pedestrians, such as children, elderly individuals, or people with disabilities. As a result, detection models may perform unevenly across different demographic groups, leading to systematic biases and potential safety risks in real-world scenarios.

While sensor fusion enhances system performance, it also introduces increased computational complexity and cost, often requiring advanced and resource-intensive methods [153]. This may limit the accessibility of such technologies to well-funded organizations, raising concerns about fairness and the equitable deployment of safety-critical systems.

Therefore, addressing both technical limitations and ethical implications, including dataset bias and accessibility, is important for developing reliable, safe, and fair pedestrian detection systems.

### 5.5. BEV-Based and Unified Perception Frameworks

One direction worth tracking is the shift away from image-plane detection toward BEV and unified perception frameworks. Camera detectors work in the perspective view of a single sensor, and that choice carries real costs: depth has to be inferred indirectly, occlusions are hard to reason about, and fusion with LiDAR or radar requires non-trivial projection between coordinate systems. BEV-based methods sidestep these by projecting multi-camera or multi-sensor input into a top-down grid, either through learned depth estimation or through explicit geometric transformation [154]. The resulting representation lives in the same planar geometry that downstream planning and control already use, which makes the rest of the stack simpler. For dense urban scenes, where pedestrian detection matters most, BEV representations have been reported to improve localization accuracy and to make camera–LiDAR–radar fusion more tractable [129].

Unified perception frameworks come at the same problem from a different angle. Detection, segmentation, tracking, and motion prediction have historically been implemented in separate modules, each trained on its own loss and then chained together at inference. Joint end-to-end architectures share features across these tasks and let the network exploit dependencies that a strictly modular pipeline cannot [155]. For pedestrian perception this matters in a specific way: pedestrian intent is a function of position and motion over time, not of any one frame, so jointly training detection with trajectory prediction lines up with how the problem is actually structured.

Neither paradigm is ready for embedded deployment yet. BEV inference is expensive in practice. Joint training brings its own difficulty: gradients from different tasks compete, and getting useful behaviour out of a shared backbone usually requires either careful loss balancing or task-specific scheduling [156]. BEV projections also depend on accurate extrinsic calibration and reasonable monocular depth, both of which drift in production. Progress on these—hardware-aware architecture design, calibration that survives the road, and learned depth uncertainty—is what the next round of ADAS perception work will probably be measured against.

### 5.6. XAI in Pedestrian Detection

As deep learning models grow more complex, transparency becomes increasingly important for both user trust and regulatory compliance. This means creating XAI methods that show how these models make decisions, especially in situations involving vulnerable road users [157]. These methods can help find and fix biases, make models stronger, and give important feedback for improving detection systems, which helps people trust autonomous systems in cities. XAI also helps with debugging and testing, letting developers see why a model might make mistakes, like confusing a shadow for a pedestrian or missing someone who is partly hidden.

A more ambitious direction is neuro-symbolic approaches to pedestrian-intent prediction. Rather than asking a black-box model whether a pedestrian will cross, these methods combine learned perception with explicit symbolic reasoning over pedestrian state (gaze, body orientation, position relative to the curb, time-to-arrival) to produce predictions that are both more interpretable and easier to audit [157]. Temporal feature extraction over pedestrian motion history is closely related: a model that bases its crossing prediction on a tracked motion sequence is inherently more inspectable than one reading a single frame. Whether these approaches will scale to production-grade detectors or remain useful mainly for the prediction layer is one of the open questions in the field.

## 6. Conclusions

This review surveys pedestrian detection techniques for ADAS, with attention to robust algorithms, multi-sensor fusion, and explainability. The evolution of pedestrian detection research reflects a transition from handcrafted feature engineering to data-driven and multi-modal perception systems. However, achieving reliable real-world performance in ADAS remains a system-level challenge that extends beyond model accuracy.

Future work should focus on efficient architectures, adaptive sensor fusion, and standardized evaluation protocols. The existing advancements should be integrated with real-time behavioral prediction and intent recognition to enable proactive decision-making in autonomous vehicles, especially considering the unpredictable nature of pedestrian movements [151,161]. Moreover, continued efforts are needed to address persistent challenges such as false negatives in diverse environmental conditions and the computational demands of advanced detection models [162]. Models that generalize across geographical and infrastructural contexts, as well as mitigating the impact of occlusions, remain significant hurdles [4,110]. Future work must address both technical performance and questions of explainability, fairness, and regulatory transparency to build public trust and ensure regulatory compliance [159,163]. Neuro-symbolic methods are a promising direction for interpretable pedestrian-intent prediction. Additionally, exploring more complex strategies for feature extraction, including those that encapsulate a pedestrian’s historical behavior over time, will be vital for developing highly accurate and interpretable crossing action predictors [159]. Therefore, future progress should be measured not only by lower miss rates on standard benchmarks, but by validated performance under occlusion, adverse weather, calibration drift, embedded latency constraints, and safety-critical downstream decision requirements.

## Figures and Tables

**Table 1:** Taxonomy of deep learning pedestrian detection architectures.

Taxonomy Axis	Detector Type	Representative Models	Accuracy vs. Speed	ADAS Suitability	Key Trade-Off/Limitation
Stage architecture	Two-stage	R-CNN, Fast R-CNN, Faster R-CNN, Mask R-CNN [6,37,38]	High accuracy; precise region proposals	Limited; sequential pipeline too slow for embedded hardware [39,40]	High accuracy at cost of computational overhead; unsuitable for real-time systems [39]
	One-stage	YOLO, SSD [41]	Reduced accuracy on small or densely packed objects [37,42]	Strong; used in ADAS perception	Speed–accuracy trade-off; high frame rates critical for real-time safety [43,44]
Localization strategy	Anchor-based	Faster R-CNN, SSD [6,45]	Established baseline accuracy	Moderate	Requires parameter tuning; struggles when object shapes differ [46]
	Anchor-free	CenterNet, FCOS [47,48]	Improved for small or densely distributed objects	Strong in crowded urban environments	Reduces hyperparameter tuning and increases flexibility; simplified pipeline [47]
Feature extraction paradigm	CNN-based	Standard CNN detectors [49]	High; dominant architecture; efficient local feature extraction	Strong; current standard	Captures local context only; limited ability to model long-range relationships between objects [49]
	Transformer-based	DETR and variants [49,50]	High contextual reasoning via global attention [51]	Limited; self-attention cost restricts real-time ADAS use [51]	Hybrid CNN–transformer architectures for balancing accuracy and efficiency [52]
Output representation	Bounding box	Standard object detectors	Approximate localization	Strong; standard ADAS approach; lower compute	Less precise localization; adequate for most ADAS scenarios [53]
	Instance segmentation	Mask R-CNN [38,54]	Pixel-level precision; beneficial in occlusion/dense environments	Limited; higher compute requirement [53]	Must balance detailed segmentation with real-time inference needs [53]
Computational profile	Lightweight	Tiny-YOLO, MobileNet-SSD [7,55]	Moderate; accuracy traded for efficiency	Strong; designed for resource-constrained embedded ADAS platforms	Accuracy trade-off; optimized for fast inference and reduced memory on edge devices [56,57]
	Heavyweight	Full-scale YOLO variants, transformer-based detectors [55]	High detection accuracy	Limited; significant compute impedes embedded use [56,57]	Timely pedestrian detection essential for safe operation; compute cost limits deployment [56]

**Table 2:** Comparative literature summary of representative RGB-only pedestrian detection methods on the Caltech and CityPersons benchmarks. log-average Miss Rate (MR^−2)^ is the log-average Miss Rate (%, lower is better) over the False Positives Per Image (FPPI) range $\left[10^{-2},\;10^{0}\right]$ on the Reasonable (R) and Heavy Occlusion (HO) subsets of CityPersons (CP).

Technique	Method	Year	Taxonomy Category	Params (M)	Input	Caltech	CP (R)	CP (HO)	Approx. Speed	Hardware	ADAS Suit.	Ref.
Classical Handcrafted-Feature Methods	HOG + linear SVM	2005	Sliding window + linear SVM	N/A	multiscale	68.0	N/E	N/E	∼1 FPS (L)	CPU, single-thread	Reference	[8,20]
	Viola–Jones	2003/2004	Haar features + AdaBoost	N/A	multiscale	94.7	N/E	N/E	≪1 FPS (L)	700 MHz Pentium III	Reference	[8,21,58]
	ACF	2014	Channel features + boosting	N/A	multiscale	32.2	N/E	N/E	∼30 FPS (L)	Single CPU core	Reference	[22]
	LDCF	2014	Locally decorrelated channels + AdaBoost trees	N/A	multiscale	24.8	N/E	N/E	∼5–15 FPS (L)	Single CPU core	Reference	[25]
	Checkerboards	2015	Filtered channels + boosting	N/A	multiscale	18.5	N/E	N/E	∼1–5 FPS (L)	Single CPU core	Reference	[24]
	DPM (LatSvm-V2)	2010	Part-based model + SVM	N/A	multiscale	63.3	N/E	N/E	∼2 FPS (L)	Single CPU core	Reference	[8,26,27]
Two-Stage Deep Learning Detectors	Faster R-CNN (vanilla)	2015	Anchor-based/CNN	∼137	∼600 px	∼21	∼15	N/E	∼5 FPS (L)	NVIDIA Tesla K40	Limited	[6,59]
	Adapted Faster R-CNN	2017	Anchor-based/CNN	∼137	×1.3	∼5.0	12.97	50.5	∼5 FPS (L)	NVIDIA Titan X	Limited	[59]
	Mask R-CNN	2017	Instance segm./CNN	∼44	∼800 px	N/E	N/E	N/E	∼5 FPS (L)	NVIDIA Tesla M40	Limited	[38]
One-Stage Deep Learning Detectors	SSD (300 × 300)	2016	Anchor-based/CNN	∼26.3	300 × 300	N/E	N/E	N/E	∼59 FPS (L)	NVIDIA Titan X	Moderate	[45]
	YOLOv5s	2020	Anchor-based/CNN	∼7.2	640 × 640	N/E	N/E	N/E	∼140 FPS (T, batch = 32)	NVIDIA Tesla V100	Moderate	[43,55]
	YOLOv7	2022	E-ELAN backbone + anchor-based head	∼36.9	640 × 640	N/E	N/E	N/E	∼83 FPS (L)	NVIDIA Tesla V100	Moderate	[60]
	YOLOv8s	2023	Anchor-free/CNN	11.17	640 × 640	N/E	∼13.0	N/E	∼91 FPS (L)	RTX 3060/3070	High	[37,55]
	HF-YOLO	2024	CNN + multi-scale feature fusion	N/R	N/R	N/E	N/E	N/E	∼45 FPS (L)	N/R	Moderate	[61]
	RetinaNet	2017	Anchor-based/focal loss	∼37.7	∼800 px	N/E	14.6	N/E	∼5 FPS (L)	NVIDIA M40	Limited	[8]
Anchor-Free Detectors	CSP (Center & Scale Pred.)	2022	Anchor-free/CNN	N/R	×1.3	4.5	11.0	∼49.3	∼22 FPS (L)	NVIDIA GTX 1080 Ti	Limited	[47]
	FCOS (R50-FPN)	2019	Anchor-free/CNN	∼32	∼800 px	N/E	N/E	N/E	∼22 FPS (L)	NVIDIA Tesla V100	Limited	[62]
Transformer-Based Detectors	DETR (ResNet-50)	2020	End-to-end/transformer	∼41	∼800 px	N/E	N/E	N/E	∼28 FPS (L)	NVIDIA Tesla V100	Limited	[50]
	RT-DETRv2 (R50)	2024	Hybrid CNN–transformer	∼42	640 × 640	N/E	N/E	N/E	∼108 FPS (T, TensorRT)	NVIDIA T4	Moderate	[63,64]
Lightweight/Edge-Deployment Models	Tiny-YOLOv3	2018	Lightweight/CNN	∼8.7	416 × 416	N/E	N/E	N/E	∼21–45 FPS (L)	NVIDIA Jetson Nano	High	[65]
	YOLOv8n	2023	Lightweight/anchor-free	3.16	640 × 640	N/E	N/R	N/E	∼1010 FPS (T, TensorRT)	NVIDIA A100	Moderate	[55]
	MobileNet-SSD	2017	Lightweight/depthwise CNN	∼6.8	300 × 300	N/E	N/E	N/E	∼60 FPS (L)	Embedded GPU	Moderate	[7,66]

Notes:Params: nominal trainable parameters (millions; may vary with class count and implementation). Speed type: L, single-image latency; T, batched or TensorRT throughput. The suitability rating reflects embedded-deployment feasibility. N/A: not applicable; N/E: not evaluated on this benchmark in the cited source; N/R: not reported.

**Table 3:** Multispectral/RGB-thermal pedestrian detection methods. These methods require aligned RGB+infrared input and are therefore evaluated on KAIST, LLVIP, or MSDS. Reported metrics differ across multispectral benchmarks.

Method	Year	Architecture	Headline Result	Ref.
KAIST baseline (ACF+T+THOG)	2015	Multispectral ACF (color + thermal channels) + AdaBoost trees	54.4% MR^−2^ on KAIST All-Day reasonable subset; runs at ∼5–8 FPS on a single CPU core	[67]
TFDet (RGB-T)	2024	Two-stream VGG-16 Faster R-CNN with target-aware fusion (FFM + FRM modules + correlation-maximum loss)	<5% MR^−2^ on KAIST All-Day reasonable; first multispectral method below 5% MR; reported as state-of-the-art at publication	[44]
PedDet (spectral optim.)	2025	Two-stream YOLOv10 with adaptive multi-scale spectral feature perception (MSFPM) and illumination robustness feature decoupling (IRFDM)	+6.6% absolute mAP gain over previous state-of-the-art on LLVIP and MSDS benchmarks	[68]

**Table 5:** Benchmark datasets used for pedestrian detection research.

Dataset	Year	Category	Sample	Sensor Modality	Key Characteristics
Caltech Pedestrian [132]	2009	Pedestrian-specific	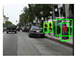	RGB video (vehicle-mounted)	Extensive video sequences in urban environments; annotations under varying scales, occlusion levels, and motion patterns; foundational benchmark
CityPersons [59,133]	2017	Pedestrian-specific	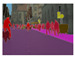	RGB image	Built on Cityscapes; high-quality bounding box annotations; dense crowds, partial occlusions, and diverse viewpoints; commonly used in ADAS scenarios
KITTI [134]	2012	Multi-sensor	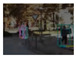	Stereo RGB, LiDAR	Synchronized stereo camera and LiDAR data; urban, rural, and highwayscenes; scale variation, motion blur, and partial occlusion; links academic research to real-world ADAS evaluation
EuroCity Persons [135]	2019	Pedestrian-specific	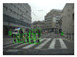	RGB image	Data from several European cities; various weather and lighting conditions; standard benchmark for robustness testing in real driving scenarios
WiderPerson [136]	2019	Pedestrian-specific	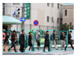	RGB image	Crowded scenes with heavy occlusion; challenging benchmark for modern pedestrian detection models
KAIST Multispectral [67]	2015	Multispectral	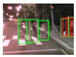	RGB, thermal infrared	Aligned visible and thermal infrared image pairs; enables evaluation of multispectral fusion strategies; especially useful for nighttime and poor-lighting conditions
CVC-14 [137]	2016	Multispectral	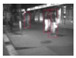	RGB, thermal infrared	Aligned visible and thermal infrared sequences; day and night subsets; testing pedestrian detection under varying lighting conditions
nuScenes [131]	2020	Multi-sensor (autonomous driving)	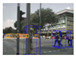	RGB, LiDAR, radar, GPS, IMU	Wide range of multi-sensor data; broad sensor coverage and diverse driving conditions; useful for multi-sensor perception research; not pedestrian-specific
AIODrive [138]	2021	Multi-sensor (autonomous driving)	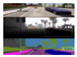	RGB, LiDAR, radar, GPS, IMU	Synchronized multi-sensor data; broad sensor coverage; useful for evaluating multi-sensor perception systems; not pedestrian-specific
Oxford Radar RobotCar [139]	2020	Multi-sensor (autonomous driving)	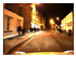	RGB, LiDAR, radar, GPS, IMU	Synchronized multi-sensor data; broad sensor coverage; useful for evaluating multi-sensor perception systems; not pedestrian-specific

**Table 6:** Open challenges and future research directions in pedestrian detection for ADAS.

Challenge	Cause	Mitigation Approaches	Open Research Directions
Adversarial attacks and robustness	Small input perturbations can cause deep detectors to misclassify in ways that matter for safety [146]	Adversarial training; certified-robust architectures; evaluation protocols that go beyond clean-data benchmarks [146]	Physically realisable adversarial scenarios for ADAS; safety certification under bounded perturbation; integration with runtime monitoring [147]
Real-time processing and efficiency	Embedded ADAS hardware has tight compute and power budgets, forcing a trade-off between accuracy, latency, and power draw [148]	Lightweight backbones; quantised and pruned inference; event-based vision for low-latency input [141,149]	Hardware-aware neural architecture search; transformer designs that survive automotive SoC constraints; co-design of perception models and inference accelerators [9]
Detection in adverse weather	Rain, snow, fog, and glare wash out edges and lower contrast, which pushes miss rates up in vision-only systems [150]	RGB–thermal multispectral fusion; illumination- and temperature-aware networks [9]; weather-augmented training [53]; event cameras for high dynamic range [141]	Adaptive fusion that re-weights modalities by scene conditions and per-prediction confidence; domain adaptation from synthetic to real adverse-weather data [9,141]
Dataset bias and demographic fairness	Benchmarks under-represent night-time scenes, children, older pedestrians, people with disabilities, and certain skin tones, so accuracy varies across groups [151,152]	Balanced dataset construction; per-subgroup evaluation; thermal imaging to reduce illumination-driven disparities [151]	Bias-aware training pipelines and standardized fairness metrics; cost-accessible perception stacks so that safety improvements do not concentrate in well-funded deployments [153]
BEV and unified perception frameworks	Image-plane detection has to infer depth indirectly and is poorly suited to occlusion reasoning; task-specific pipelines miss cross-task dependencies that matter for downstream planning [154]	Top-down BEV grids built from multi-camera or multi-sensor input; end-to-end architectures that jointly handle detection, segmentation, tracking, and motion prediction [129,155]	Making BEV inference fit automotive latency budgets; balancing task losses under joint training; keeping BEV projections stable under calibration drift and noisy depth [154,156]
Explainability (XAI)	Deep detectors behave as black boxes, which makes debugging, regulatory review, and operator trust harder [157]	Saliency- and attribution-based explanations; failure-mode diagnosis tools [158]	Neuro-symbolic methods for interpretable pedestrian-intent prediction; temporal feature extraction over pedestrian motion history for interpretable crossing-action predictors [159]

## Data Availability

No new data were created or analyzed in this study. Data sharing is not applicable to this article.

## Abbreviations

The following abbreviations are used in this manuscript:

ACF	Aggregated Channel Features
ADAS	Advanced Driver Assistance Systems
AI	Artificial Intelligence
ATSS	Adaptive Training Sample Selection
BEV	Bird’s-Eye View
CNN	Convolutional Neural Network
CSP	Center and Scale Prediction
DETR	DEtection TRansformer
DPM	Deformable Part Model
FCOS	Fully Convolutional One-Stage object detection
FPPI	False Positives Per Image
FPS	Frames Per Second
GPS	Global Positioning System
HOG	Histogram of Oriented Gradients
ICF	Integral Channel Features
IMU	Inertial Measurement Unit
KAIST	KAIST Multispectral Pedestrian Dataset
LBP	Local Binary Patterns
LDCF	Locally Decorrelated Channel Features
LiDAR	Light Detection And Ranging
LLVIP	Low-Light Visible-Infrared Paired dataset
mAP	mean Average Precision
MR^−2^	log-average Miss Rate (over FPPI ∈ [$10^{-2},\;10^{0}$])
NMS	Non-Maximum Suppression
PVT	Pyramid Vision Transformer
R	Reasonable subset (CityPersons benchmark)
R-CNN	Region-based Convolutional Neural Network
RGB	Red, Green, Blue
RoI	Region of Interest
RPN	Region Proposal Network
RT-DETR	Real-Time DEtection TRansformer
SIFT	Scale-Invariant Feature Transform
SSD	Single Shot MultiBox Detector
SURF	Speeded-Up Robust Features
SVM	Support Vector Machine
XAI	Explainable Artificial Intelligence
YOLO	You Only Look Once
